# Development of a predictive model for high-risk infertility based on TCM constitution and syndrome: a secondary analysis

**DOI:** 10.3389/fmed.2026.1738707

**Published:** 2026-02-18

**Authors:** Zhuwei Gao, Xiaoling Miao, Jiaxing Feng, Tingxian Yang, Yixiao Hu, Baichao Shi, Muxin Guan, Yu Liu, Zengtiantian He, Xiaoke Wu

**Affiliations:** 1 Heilongjiang University of Chinese Medicine, Harbin, Heilongjiang, China; 2 Yunnan University of Chinese Medicine, Kunming, Yunnan, China; 3 First Affiliated Hospital, Heilongjiang University of Chinese Medicine, Harbin, Heilongjiang, China; 4 Kunming Maternal and Child Health-care Hospital, Kunming, Yunan, China; 5 Qujing Maternal and Child Health-Care Hospital, Qujing, Yunnan, China; 6 Zhejiang Provincial Hospital of Chinese Medicine, Zhejiang Chinese Medical University, Hangzhou, Zhejiang, China

**Keywords:** high risk, infertility, predictive model, TCM constitution types, TCM syndrome

## Abstract

**Objective:**

To evaluate the informational contribution of Traditional Chinese Medicine (TCM) variables to high-risk infertility stratification and to develop predictive models integrating TCM and Western medical factors.

**Methods:**

Data from 409 women with infertility were analyzed. High-risk infertility was defined as infertility duration >3 years or the presence of ≥2 documented infertility-related causes. Demographic, reproductive, menstrual, and Traditional Chinese Medicine (TCM) constitution and syndrome variables were collected. Three predictive models— a TCM model (Model 1), a modern medicine model (Model 2), and an integrated model (Model 3)—were developed using independent candidate variable sets for each model. Variable selection was performed separately for each model using Least Absolute Shrinkage and Selection Operator (LASSO) logistic regression with 10-fold cross-validation. Multivariable logistic regression models were then fitted based on the selected predictors. Model performance was assessed using Receiver Operating Characteristic (ROC) analysis, calibration curves, and decision curve analysis (DCA). Internal validation and optimism correction were conducted using bootstrap resampling with an out-of-bag strategy.

**Results:**

Among the participants, 163 women (39.9%) were classified as high-risk infertility. The high-risk group was older and differed significantly in reproductive history and clinical characteristics. The Model 3 showed a slight improvement in apparent discrimination compared with the Model 2 [area under the curve (AUC) = 0.790 vs. 0.771]. However, after optimism correction, the Model 2 demonstrated more robust discrimination, calibration, and overall predictive accuracy. The Model 3 exhibited miscalibration after correction, indicating the need for further shrinkage and recalibration. The Model 1 showed limited discriminative ability (AUC = 0.606).

**Conclusion:**

After internal validation, the modern medicine model showed the most stable predictive performance. The integrative model mainly contributed to exploring the potential informational value of TCM variables in infertility risk stratification rather than improving predictive accuracy. These findings provide methodological support for infertility risk assessment within an integrative Chinese and Western medicine framework.

## 1 Introduction

Infertility is defined as the failure to conceive after 12 months of regular unprotected sexual intercourse. Globally, an estimated 186 million individuals are affected by infertility, making it a significant public health concern worldwide (1). The three primary determinants of infertility are disease duration, female age, and disease-related infertility. The likelihood of natural conception decreases with prolonged infertility (2). In clinical practice, a subset of patients exhibits prolonged disease courses, complex etiologies, low probability of spontaneous pregnancy, and unclear treatment strategies. These patients are often considered to be in a “high-risk” state. However, there is currently no standardized criterion for defining high-risk infertility, leading to challenges in determining the appropriate timing for fertility interventions. Moreover, clinicians lack structured prediction tools to support decision-making regarding treatment initiation and stratified management. Previous studies have shown that individualized risk prediction models can help patients make more informed decisions between “watchful waiting” and “active intervention,” thereby optimizing treatment strategies and reducing unnecessary healthcare costs and psychological burden (3).

TCM constitution and syndrome are regarded as key diagnostic frameworks for understanding individual susceptibility and disease progression. Prior studies have demonstrated that TCM constitution is not only associated with metabolic indicators such as hypertension and fasting glucose but also exhibits a stable genetic basis, with specific constitution types identifiable through gene expression profiling with high accuracy (4, 5). TCM syndromes, on the other hand, are considered functional imbalance patterns corresponding to different disease stages and trajectories and have been shown to have biological correlates in conditions such as rheumatoid arthritis (6). These findings provide both theoretical rationale and empirical support for the application of constitution and syndrome in disease risk prediction and individualized intervention. In recent years, advances in the objectification of TCM diagnostics have sparked growing interest in integrating TCM features into modern prediction models to assess disease risk. However, empirical modeling studies specifically focused on infertility remain limited.

This study was based on clinical data from 409 women with infertility. High-risk infertility was defined as an infertility duration longer than three years or the presence of two or more clearly identified infertility-related etiologies. By systematically integrating multidimensional information—including demographic characteristics, lifestyle factors, reproductive and contraceptive history, as well as TCM constitution and syndrome patterns—we constructed and evaluated three types of prediction models: a TCM model containing only TCM diagnostic variables (Model 1), a modern medicine model including only demographic and contemporary clinical variables (Model 2), and an integrative model combining both TCM and modern medical factors (Model 3). This design allowed comparison of the predictive contributions from different information sources and exploration of the independent and complementary value of TCM diagnostic information in infertility risk stratification. It should be emphasized that the definition of “high-risk infertility” in this study was used solely as an analytical endpoint for model development and validation, rather than as a substitute for clinical diagnostic criteria. Although the models were trained using a clearly defined high-risk outcome, their intended application is not limited to women who already meet this definition. The developed predictive models are not intended as confirmatory tools after a definitive diagnosis, but rather to support structured risk stratification during the clinical evaluation of infertility. They are particularly applicable to women whose etiological assessment is still ongoing but who already exhibit multidimensional high-risk characteristics, thereby facilitating early identification, intensified follow-up, and individualized intervention. The analytical framework of this study centers on predictive modeling, with the primary objective of evaluating the discriminative ability and potential clinical utility of different variables and models for risk identification, rather than inferring causal relationships or elucidating the biological mechanisms underlying infertility.

## 2 Materials and methods

### 2.1 Data source

This study is a secondary analysis of existing clinical data, derived from an original research project previously published (7). The original dataset was obtained from a cross-sectional questionnaire survey conducted among infertile women attending the gynecology outpatient clinic of Yunnan Provincial Hospital of TCM between January 1 and December 31, 2018. The survey employed a structured questionnaire administered by uniformly trained investigators during face-to-face interviews. Collected information included basic demographic characteristics, reproductive history, Western medical diagnoses, and assessments of TCM syndromes and constitution types. During routine clinical visits, infertility evaluations were performed in accordance with standard clinical practice, typically including detailed reproductive history-taking, gynecological examination, pelvic ultrasonography, and basic endocrine testing. Infertility-related etiologies were defined based on diagnoses explicitly recorded and confirmed by attending clinicians in the medical records, and were subsequently counted once a definitive diagnosis had been established. To ensure data quality, cases with substantial missing information or incomplete key diagnostic data were excluded. After data screening, a total of 409 infertile women were included in the final analysis.

Inclusion criteria were as follows: (1) married women with a desire for childbirth; (2) meeting the diagnostic criteria for infertility as described above; and (3) willingness and ability to complete clinical data collection. Exclusion criteria included: (1) inability to cooperate or poor compliance (e.g., psychiatric disorders); (2) infertility attributable to male factors; and (3) incomplete data due to any reason.

### 2.2 Definitions and diagnostic criteria

The diagnosis of infertility was based on the criteria outlined in Gynecology and Obstetrics (10th Edition, People’s Medical Publishing House, 2024) (8). TCM constitution types were determined according to the 2009 classification guidelines issued by the China Association of Chinese Medicine, titled “Classification and Determination of TCM Constitution (9). This framework categorizes the constitution into nine types: Balanced constitution, Yang-deficiency constitution, Qi-depression constitution, Damp-heat constitution, Qi-deficiency constitution, Yin-deficiency constitution, Phlegm-dampness constitution, Blood-stasis constitution, and Special constitution. TCM syndrome differentiation was performed based on four classical patterns from the textbook Gynecology of TCM: Kidney-deficiency syndrome, Liver-depression and Qi-stagnation syndrome, Stasis obstruction in the uterus syndrome, Phlegm-dampness obstruction syndrome, and Others. Cases that could not be classified into one of these four types—such as combined syndromes like Kidney-deficiency with blood stasis, or Qi stagnation with blood stasis—were uniformly categorized as “other syndromes” and included as an independent variable in model construction.

### 2.3 Variable selection and model construction

#### 2.3.1 Definition of high-risk infertility and outcome construction

Several clinical guidelines recommend that women with an infertility duration of >3 years undergo early laparoscopic evaluation or be considered for assisted reproductive technologies (10–12). Meanwhile, studies have shown that approximately 93% of women can achieve spontaneous pregnancy within three years, and a disease duration beyond this threshold indicates potential underlying etiologies or a significantly reduced probability of natural conception (12). Currently, there is no standardized criterion for stratifying risk in “multi-etiological infertility.” However, in clinical settings, the coexistence of multiple confirmed infertility-related factors often implies multifaceted dysfunction within the reproductive system, complicating both the path to pregnancy and intervention strategies. Accordingly, this study defined “high-risk infertility” as the primary outcome variable, operationalized as meeting either of the following criteria: (1) an infertility duration of >3 years; or (2) the presence of two or more diagnosed infertility-related etiologies (e.g., coexisting ovarian and tubal factors). This definition was designed to identify a clinically representative population characterized by prolonged disease course, complex causes, unclear treatment paths, and low likelihood of spontaneous conception—hallmarks of the high-risk infertile state. To avoid incorporating potential predictor variables into the outcome definition—thereby reducing the risk of reverse causality or confounding bias—variables such as age, constitution type, and lifestyle factors were excluded from the definition of high-risk infertility. However, they were instead treated as independent variables during model construction.

#### 2.3.2 Candidate variable construction and data preprocessing

High-risk infertility status was defined as the dependent variable. Based on clinical availability and prior evidence, an initial set of candidate predictors was constructed, including demographic characteristics (age, body mass index, occupation, and education level), reproductive and contraceptive history (gravidity, parity, number of previous miscarriages, history of gynecological surgery, and contraceptive method), menstrual characteristics (age at menarche, menstrual cycle length, menstrual duration, and menstrual volume), and Traditional Chinese Medicine–related features (TCM syndromes and TCM constitution types). Before formal statistical analyses, the extent of missing data in candidate predictors was assessed (Supplementary Table S1). Given the overall low proportion of missingness (≤2.7% for most variables, with a maximum of 8.6% for age at menarche), single imputation was applied during the model-building stage to generate a complete analysis dataset. Specifically, continuous variables (e.g., age and body mass index) were imputed using the sample median, while categorical and ordinal variables (e.g., occupation, age at menarche, and menstrual duration) were imputed using the mode. Multivariable predictive model development and internal validation were then conducted using the imputed complete dataset (*N* = 409). For univariate comparisons of baseline characteristics, complete-case analysis was applied to minimize the potential influence of imputed values on descriptive statistics. Accordingly, only observations without missing values for the variable under comparison were included, resulting in variable-specific effective sample sizes. All categorical variables were incorporated into the analyses using dummy-variable encoding.

#### 2.3.3 Variable selection and predictive model development

After candidate variable construction and preprocessing, predictive models were developed using three distinct sets of candidate variables to evaluate the predictive performance of different variable combinations for high-risk infertility. Specifically, these included: Model 1 (TCM model), which incorporated only TCM-related variables (TCM syndromes and TCM constitution types); Model 2 (modern medicine model), which included clinically available variables other than TCM variables (including demographic characteristics, reproductive and contraceptive history, menstrual-related characteristics, and lifestyle-related information); and Model 3 (integrated model), which simultaneously incorporated TCM variables and modern medical variables to comprehensively reflect the potential predictive value of multidimensional information for high-risk infertility. These three models were constructed independently within their respective prespecified candidate-variable domains, with no shared variable-selection procedures across models.

All three models employed LASSO logistic regression for variable selection and model development. The optimal penalty parameter (*λ*_min) was determined using 10-fold cross-validation, and variables with non-zero regression coefficients at this λ value were retained as candidate predictors. Subsequently, separate binary logistic regression models were fitted using the selected variables to estimate the individual probability of high-risk infertility and to evaluate overall model performance. For each model, variable selection, penalty tuning, and subsequent logistic regression fitting were conducted independently within the corresponding variable set. The primary objective of model development in this study was risk prediction and performance assessment, rather than inference on causal relationships or independent effects of individual variables.

#### 2.3.4 Model performance evaluation and validation

A multidimensional set of performance metrics was used to comprehensively evaluate the three predictive models, including discrimination, calibration, and clinical net benefit. Discrimination was assessed using ROC curves and the AUC. The 95% confidence intervals for the AUC were estimated using 2,000 nonparametric bootstrap resamples to quantify within-sample uncertainty in discriminative performance. Calibration performance was evaluated by calibration curves to visually assess agreement between predicted probabilities and observed event rates. Bootstrap resampling was applied to estimate and correct for optimism in model performance. Specifically, 1,000 bootstrap resamples were used to generate optimism-corrected calibration curves, with calibration intercepts, calibration slopes, and Brier scores reported to quantitatively characterize systematic bias and overall prediction error.

To further assess model generalizability and reduce performance inflation arising from model development and evaluation on the same dataset, an internal validation procedure based on the bootstrap out-of-bag (OOB) principle was applied. In each bootstrap iteration, the predictive model was fitted on the resampled dataset and evaluated on the corresponding OOB observations to obtain performance metrics, including AUC, Brier score, calibration intercept, and calibration slope. Results were aggregated across resamples to obtain optimism-corrected estimates of model discrimination and prediction error. In the present study, 200 bootstrap–OOB resamples were performed. It should be noted that the bootstrap–OOB internal validation was conducted using a fixed set of predictors determined after LASSO-based variable selection on the full dataset. Variable selection and tuning of the penalty parameter (*λ*) were not repeated within each bootstrap resample. Therefore, the reported corrected performance metrics primarily reflect the stability of model fitting and prediction under a fixed model structure and do not fully account for optimism introduced during the variable selection and tuning stages.

Clinical utility was evaluated using DCA, comparing net benefit across threshold probabilities ranging from 0 to 0.9. For visualization and clinical interpretability, nomograms were constructed based on the final fitted models, mapping predictor-specific point scores to individualized predicted probabilities of high-risk infertility. All performance metrics and nomograms were derived from the independently constructed and optimized final models, with variables included in each nomogram fully corresponding to those in the respective predictive model.

### 2.4 Statistical analysis

This study was conducted using R software version 4.5.0 (R Foundation for Statistical Computing, Vienna, Austria) in combination with the RStudio platform for data management and statistical analysis. Prior to model development, all variables were screened for missing values. Variables with low missing-data rates were handled using complete-case analysis. The distributional characteristics of continuous variables were assessed before analysis. As most variables did not meet the assumption of normality, continuous variables were summarized as medians (interquartile ranges), and between-group comparisons were performed using the Mann–Whitney U test. Categorical variables were expressed as frequencies (percentages), and group comparisons were conducted using the Pearson chi-square test or Fisher’s exact test, as appropriate.

In the predictive modeling analyses, all multi-category variables were treated as unordered nominal variables and entered into the models using dummy-variable encoding. For categorical variables with multiple levels, a prespecified reference category was used to generate k − 1 dummy variables, thereby avoiding assumptions regarding ordinal structure or equal spacing between categories. In the regression results, only non-reference levels retained after LASSO-based variable selection were reported. Continuous variables were examined for their distributions prior to modeling. During the LASSO variable selection stage, all candidate predictors were standardized to ensure comparability across variables with different measurement scales under penalized regression.

Variable selection was performed using LASSO logistic regression, with the optimal penalty parameter (*λ*) determined by ten-fold cross-validation. Variables with non-zero coefficients in the model corresponding to λ_min were retained. The penalty was applied at the level of individual candidate features, including those generated through dummy-variable expansion, thereby enabling practical shrinkage and selection in a high-dimensional predictor space. All statistical tests were two-sided, and a *p* < 0.05 was considered statistically significant.

## 3 Results

### 3.1 Comparison of baseline characteristics between risk groups

In the 409 female infertility patients included, 163 (39.9%) were in the high-risk group, and 246 (60.1%) were in the non-high-risk group. Baseline characteristic comparisons showed that the median age of the high-risk group was significantly higher than that of the non-high-risk group [34 (31, 38) years vs. 31 (28, 35) years; difference = 3; 95% CI: 1.61–4.39; *p* < 0.001]. There was a statistically significant difference in occupational composition between the two groups (χ^2^ = 24.656, *p* = 0.00604). The distribution of sexual activity frequency also differed (χ^2^ = 3.983, *p* = 0.046), with a higher proportion of “low-frequency” sexual activity in the high-risk group [61.3% vs. 50.8%]. Among reproductive history-related indicators, the two groups differed in the number of pregnancies (*p* = 0.0482), and the high-risk group also showed a difference in the number of miscarriages (*p* = 0.0218). Additionally, the proportion of individuals with a history of gynecological surgery was significantly higher in the high-risk group compared to the non-high-risk group [39.3% vs. 24.8%, χ^2^ = 8.999, *p* = 0.0027]. There was a significant difference in the overall distribution of contraceptive methods between the two groups (χ^2^ = 20.842, *p* < 0.001), with a higher proportion of women in the high-risk group reporting “never used contraception” [50.3% vs. 31.7%], while the proportion using condoms was lower [35.0% vs. 50.8%].

No significant differences were found between the two groups for the other variables (all *p* > 0.05), including BMI, ethnicity, education level, age at menarche, menstrual characteristics (cycle, duration, volume), number of deliveries, and menstrual irregularities (Table 1).

**Table 1:** Baseline characteristics of patients across infertility risk groups.

Val	Non-high-risk (*n* = 246)	High-risk (*n* = 163)	Difference (95% CI)	Z/χ^2^	*P*
Age	31 (28,35)	34 (31,38)	3 (1.61–4.39)	−5.413	<0.001*
BMI	22.2 (20.07,24.29)	22.1 (20.3,24.44)	−0.1 (−0.93–0.73)	−0.412	0.68
Ethnicity				0	1
Han	53 (21.5%)	35 (21.5%)			
Minority	193 (78.5%)	128 (78.5%)			
Occupation				24.656	0.00604*
General employee	53 (21.5%)	29 (17.8%)			
Self-employed	32 (13%)	33 (20.2%)			
Technical staff	51 (20.7%)	26 (16%)			
Farmer	30 (12.2%)	28 (17.2%)			
Unemployed	15 (6.1%)	17 (10.4%)			
Business manager	14 (5.7%)	16 (9.8%)			
Freelancer	18 (7.3%)	9 (5.5%)			
Civil servant	20 (8.1%)	5 (3.1%)			
Worker	11 (4.5%)	0 (0%)			
Teacher	1 (0.4%)	0 (0%)			
Others	1 (0.4%)	0 (0%)			
Education level				7.965	0.0929
Junior high or below	35 (14.2%)	37 (22.7%)			
High school	52 (21.1%)	38 (23.3%)			
Junior college	50 (20.3%)	35 (21.5%)			
University	98 (39.8%)	49 (30.1%)			
Postgraduate	11 (4.5%)	4 (2.5%)			
Sexual activity frequency				3.983	0.046*
Low	125 (50.8%)	100 (61.3%)			
Normal	121 (49.2%)	63 (38.7%)			
Age at menarche				2.241	0.326
≤12 years	60 (24.4%)	35 (21.5%)			
13–14 years	155 (63%)	99 (60.7%)			
≥15 years	31 (12.6%)	29 (17.8%)			
Menstrual duration				1.739	0.419
≤2 days	8 (3.3%)	2 (1.2%)			
3–7 days	216 (87.8%)	145 (89%)			
≥8 days	22 (8.9%)	16 (9.8%)			
Cycle length				5.202	0.0742
≤20 days	0 (0%)	3 (1.8%)			
21–35 days	166 (67.5%)	114 (69.9%)			
≥36 days	80 (32.5%)	46 (28.2%)			
Menstrual volume				5.837	0.054
Light	86 (35%)	76 (46.6%)			
Normal	130 (52.8%)	73 (44.8%)			
Heavy	30 (12.2%)	14 (8.6%)			
Gravidity	1 (0.2)	1 (0.2)	0 (−0.4–0.4)	−1.901	0.0482*
Parity	0 (0.1)	0 (0.1)	0 (−0.2–0.2)	0.601	0.448
Number of miscarriages	1 (0.1)	1 (0,2)	0 (−0.33–0.33)	−2.144	0.0218*
Gynecological surgery history				8.999	0.0027*
Yes	61 (24.8%)	64 (39.3%)			
No	185 (75.2%)	99 (60.7%)			
Contraceptive method				20.842	<0.001*
IUD	23 (9.3%)	9 (5.5%)			
Emergency contraception	10 (4.1%)	5 (3.1%)			
Female sterilization	0 (0%)	3 (1.8%)			
Hormonal contraception	10 (4.1%)	7 (4.3%)			
Condom	125 (50.8%)	57 (35%)			
Never used	78 (31.7%)	82 (50.3%)			

Continuous variables are presented as median (interquartile range) and were compared between groups using the Mann–Whitney U test, with Z statistics reported. Categorical variables are presented as numbers (percentages) and were compared using the chi-square (χ^2^) test or Fisher’s exact test, with χ^2^ statistics reported. *p < 0.05.

### 3.2 Comparison of TCM syndrome patterns and constitution types between risk groups

In terms of TCM syndromes, Kidney-deficiency syndrome had the highest distribution in both groups, with a higher prevalence in the high-risk group (65.0% vs. 54.9%), and the difference approached statistical significance (*p* = 0.0523). Phlegm-dampness obstruction syndrome had a significantly lower prevalence in the high-risk group (2.5% vs. 8.9%, *p* = 0.0152), making it the only syndrome showing a statistically significant difference between the groups. Other syndromes (such as Liver-depression and Qi-stagnation syndrome, Blood-stasis syndrome, and others) showed no significant differences in distribution between the two groups (*p* > 0.05) (Table 2).

**Table 2:** Distribution of TCM syndrome patterns by infertility risk group.

Val	Non-high-risk (*n* = 246)	High-risk (*n* = 163)	Z/χ^2^	*P*
Kidney-deficiency syndrome			3.766	0.0523
No	111 (45.1%)	57 (35%)		
Yes	135 (54.9%)	106 (65%)		
Liver-depression and Qi-stagnation syndrome			0.102	0.749
No	215 (87.4%)	145 (89%)		
Yes	31 (12.6%)	18 (11%)		
Stasis obstruction in the uterus syndrome			1.129	0.288
No	225 (91.5%)	143 (87.7%)		
Yes	21 (8.5%)	20 (12.3%)		
Phlegm-dampness obstruction syndrome			5.888	0.0152*
No	224 (91.1%)	159 (97.5%)		
Yes	22 (8.9%)	4 (2.5%)		
Others			2.508	0.113
No	209 (85%)	148 (90.8%)		
Yes	37 (15%)	15 (9.2%)		

Among TCM constitution types, Qi-deficiency constitution was more common in the high-risk group (12.9% vs. 5.3%), with a statistically significant difference (*p* = 0.011), suggesting a possible association with high-risk infertility. Other constitutions (such as Yang-deficiency, Yin-deficiency, Phlegm-dampness, Damp-heat, Blood-stasis, Special constitution, Qi-stagnation, and Balanced constitution) showed no statistically significant differences in distribution between the two groups (*p* > 0.05) (Table 3).

**Table 3:** Distribution of TCM constitution types by infertility risk group.

Val	Non-high-risk (*n* = 246)	High-risk (*n* = 163)	Z/χ^2^	*P*
Yang-deficiency constitution			0.639	0.424
No	198(80.5%)	125(76.7%)		
Yes	48(19.5%)	38(23.3%)		
Yin-deficiency constitution			0	1
No	228(92.7%)	151(92.6%)		
Yes	18(7.3%)	12(7.4%)		
Qi-deficiency constitution			6.464	0.011*
No	233(94.7%)	142(87.1%)		
Yes	13(5.3%)	21(12.9%)		
Phlegm-dampness constitution			0	1
No	236(95.9%)	156(95.7%)		
Yes	10(4.1%)	7(4.3%)		
Damp-heat constitution			0	1
No	218(88.6%)	144(88.3%)		
Yes	28(11.4%)	19(11.7%)		
Blood-stasis constitution			0	1
No	239(97.2%)	158(96.9%)		
Yes	7(2.8%)	5(3.1%)		
Special constitution			0	1
No	244(99.2%)	162(99.4%)		
Yes	2(0.8%)	1(0.6%)		
Qi-stagnation constitution			2.152	0.142
No	203(82.5%)	144(88.3%)		
Yes	43(17.5%)	19(11.7%)		
Balanced constitution			1.518	0.218
No	169(68.7%)	122(74.8%)		
Yes	77(31.3%)	41(25.2%)		

### 3.3 Candidate predictor selection based on LASSO regression

**Figure 1:**
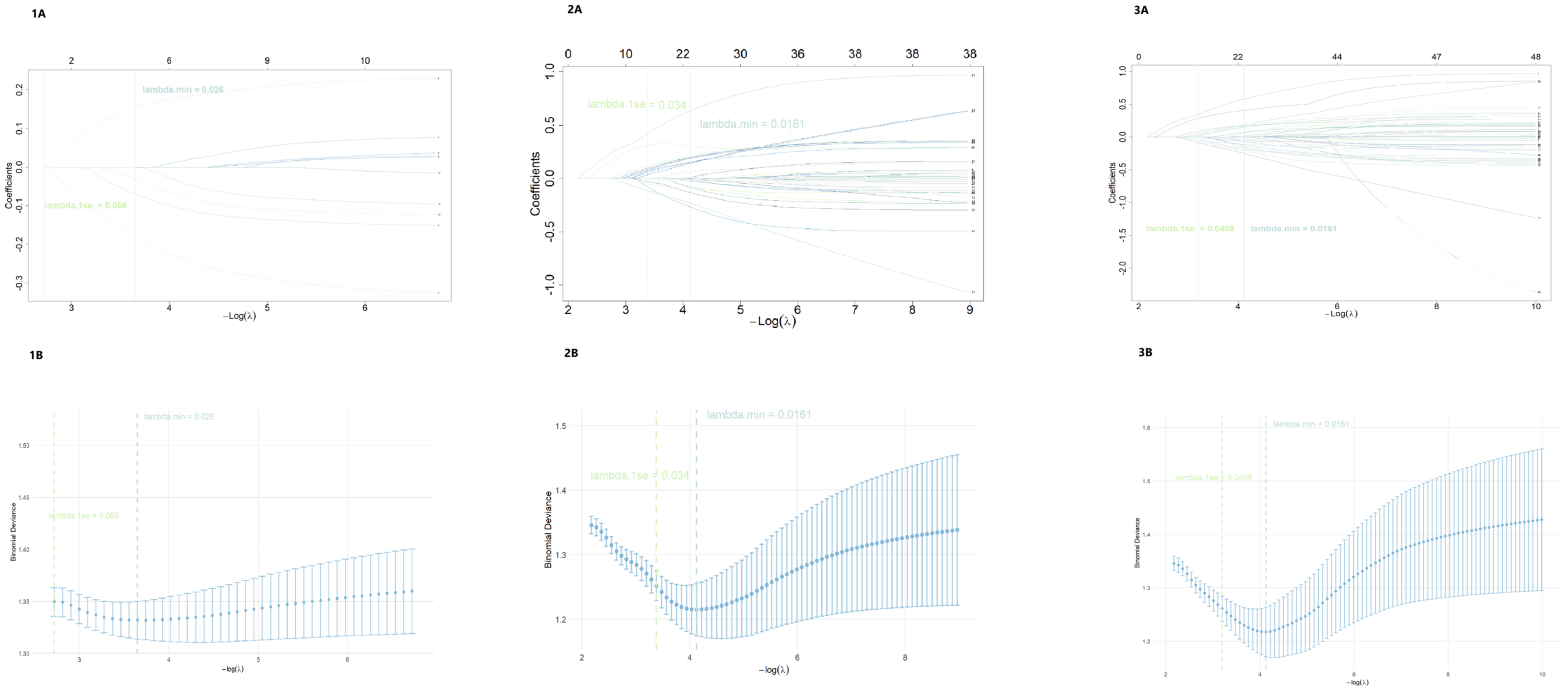
Cross-validation curve and coefficient path plot of LASSO regression. Panels 1A–3A show the LASSO coefficient path plots for Model 1, Model 2, and Model 3, respectively. The *x*-axis represents log(*λ*), and the *y*-axis represents the regression coefficients of each candidate variable. The curves illustrate the shrinkage process of the variable coefficients under different penalty parameters. Panels 1B–3B correspond to the 10-fold cross-validation curves used to determine the optimal penalty parameter λ. The dashed lines represent λ_min (the λ that minimizes cross-validation error) and λ_1se (the maximum λ within one standard error of the minimum error). The final variable selection is based on the non-zero regression coefficients corresponding to the predictors selected at λ_min.

Based on data from 409 women with infertility, LASSO logistic regression was performed to select variables for Models 1, 2, and 3 (Figure 1). In each model, the optimal penalty parameter λ_min was determined via 10-fold cross-validation, and variables with non-zero regression coefficients at λ_min were retained as candidate predictors for subsequent model construction. In Model 1, only TCM syndrome and constitution-related variables were included for selection. A total of 4 non-zero coefficient variables were selected at λ_min, including Phlegm-dampness obstruction syndrome, other syndrome, Qi-deficiency constitution, and Qi-stagnation constitution (Table 4). Among them, Qi-deficiency constitution showed a positive predictive effect.

**Table 4:** Standardized coefficients of predictors for Model 1.

Predictor	Standardized coefficient
TCM syndrome: Phlegm-dampness obstruction	−0.181
TCM syndrome: Others	−0.069
TCM constitution: Qi-deficiency	0.154
TCM constitution: Qi-stagnation	−0.031

The table lists the non-zero coefficient predictors selected based on the optimal penalty parameter (λ_min) determined through 10-fold cross-validation in LASSO Logistic regression, along with their standardized regression coefficients. A positive coefficient indicates a positive association with the probability of high-risk infertility, while a negative coefficient indicates a negative association. For categorical variables, the categories shown in the tables represent non-reference levels relative to the prespecified reference category (Supplementary Table S2).

In contrast, Phlegm-dampness obstruction syndrome, other syndrome, and Qi-stagnation constitution had negative predictive value, indicating that TCM syndromes and constitution characteristics provide valuable predictive information for high-risk infertility stratification. In Model 2, LASSO regression at λ_min selected 10 non-zero coefficient variables, mainly covering demographic characteristics, reproductive history, menstrual characteristics, and gynecological factors (Table 5). These include age, occupational status (worker or self-employed), education level (junior high school or below), frequency of sexual activity (low), menstrual volume (light), number of miscarriages, history of gynecological surgery, and contraceptive method (female sterilization or never used contraception). Among these, age (per 1-year increase), light menstrual volume, and never used contraception had relatively large standardized regression coefficients, suggesting that these factors play a significant role in high-risk infertility prediction within the modern medical variable framework. Other variables, such as occupational status (worker), education level (junior high school or below), and gynecological surgery history, also contributed significantly to prediction. In Model 3, LASSO regression at λ_min selected 22 non-zero coefficient variables (Table 6), covering multiple dimensions, including demographic characteristics, menstrual and reproductive history, gynecological surgery history, and TCM syndrome and constitution. The relative predictive weights of Model 3 are visually displayed in a forest plot (Supplementary Figure S1).

**Table 5:** Standardized coefficients of predictors for Model 2.

Predictor	Standardized coefficient
Age (per 1-year increase)	0.634
Occupation: Worker	−0.234
Occupation: Self-employed	0.186
Education level: Junior high or below	0.200
Sexual activity frequency: Low	0.188
Menstrual volume: Light	0.194
Parity (per 1-unit increase)	−0.269
Gynecological surgery history: Yes	0.212
Contraceptive method: Female sterilization	0.151
Contraceptive method: Never used	0.301

**Table 6:** Standardized coefficients of predictors for Model 3.

Predictor	Standardized coefficient
Age (per 1-year increase)	0.583
BMI (per 1 kg/m^2^ increase)	0.062
Occupation: Self-employed	0.158
Occupation: Unemployed	0.065
Occupation: Business manager	0.144
Occupation: Civil servant	−0.087
Occupation: Worker	−0.247
Education level: University	−0.039
Sexual activity frequency: Normal	−0.167
Age at menarche: ≥15 years	0.015
Menstrual volume: Normal	−0.115
Menstrual volume: Heavy	−0.149
Parity (per 1-unit increase)	−0.206
Gynecological surgery history: No	−0.190
Contraceptive method: Female sterilization	0.182
Contraceptive method: Never used	0.401
TCM syndrome: Stasis obstruction in the uterus	0.06
TCM syndrome: Phlegm-dampness obstruction	−0.175
TCM syndrome: Others	−0.062
TCM constitution: Qi-deficiency	0.167
TCM constitution: Qi-stagnation	−0.032
TCM constitution: Balanced	−0.055

### 3.4 Visualization and performance evaluation of the predictive model

**Figure 2:**
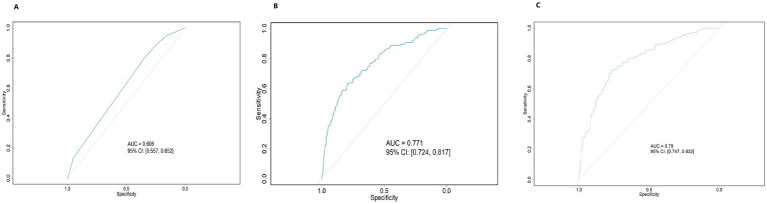
ROC curves for the prediction models of high-risk infertility. **(A)** ROC curve of Model 1; **(B)** ROC curve of Model 2; **(C)** ROC curve of Model 3.

**Figure 3:**
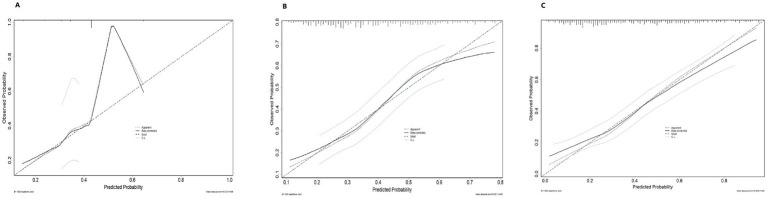
Calibration curves for the prediction models of high-risk infertility. **(A)** Calibration curve of Model 1; **(B)** Calibration curve of Model 2; **(C)** Calibration curve of Model 3.

Based on data from 409 women with infertility, three predictive models, Model 1, Model 2, and Model 3, were constructed and evaluated for their discriminative ability, calibration performance, and clinical net benefit (Table 7). In terms of apparent discriminative ability (Figure 2), Model 3 had the highest AUC (0.790), followed by Model 2 (0.771), with Model 1 having the lowest AUC (0.606). Based on the optimism-corrected results obtained from Bootstrap–OOB internal validation with 200 resamples, the corrected AUCs for Model 1, Model 2, and Model 3 were 0.588 (95% CI: 0.514–0.655), 0.744 (95% CI: 0.677–0.815), and 0.722 (95% CI: 0.648–0.786), respectively. In terms of overall prediction error, Model 3 had the lowest apparent Brier score (0.180), followed by Model 2 (0.187), and Model 1 had the highest (0.229). After optimism correction using Bootstrap–OOB internal validation, the Brier scores for the three models were 0.236 for Model 1, 0.201 for Model 2, and 0.214 for Model 3, indicating the presence of optimism in the apparent performance estimates. Calibration curves obtained using the bootstrap calibration approach (Figure 3) showed good agreement between predicted probabilities and observed risks for Model 1 and Model 2, whereas Model 3 exhibited a noticeable deviation, particularly in the higher predicted-risk range. Further Bootstrap–OOB internal validation indicated that the calibration slopes for Model 1, Model 2, and Model 3 were 0.824, 0.842, and 0.596, respectively, with corresponding calibration intercepts of −0.058, −0.039, and −0.129. The calibration slope of Model 3 was substantially lower than 1 (0.596), indicating meaningful overfitting and overly extreme predictions after the inclusion of additional predictors. These findings suggest that, under the current sample size and modeling strategy, predictions from Model 3 would require further shrinkage and recalibration prior to any prospective application.

**Table 7:** Performance comparison of high-risk infertility prediction models after bootstrap optimism correction.

Performance index	Model 1	Model 2	Model 3
Apparent AUC	0.606	0.771	0.790
Corrected AUC (95% CI)	0.588 (0.514–0.655)	0.744 (0.677–0.815)	0.722 (0.648–0.786)
Apparent brier score	0.229	0.187	0.180
Corrected brier score	0.236	0.201	0.214
Corrected calibration slope	0.824	0.842	0.596
Corrected calibration intercept	−0.058	−0.039	−0.129

**Figure 4:**
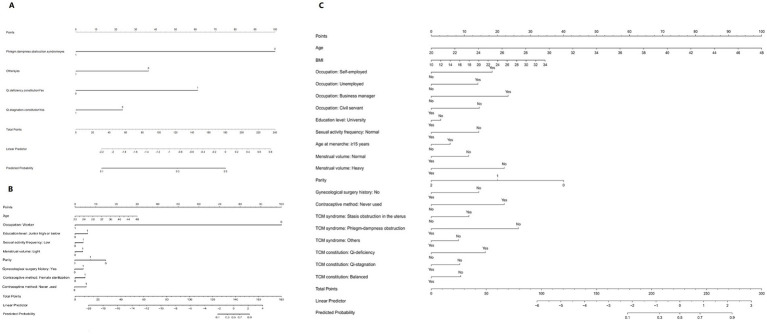
Nomograms for the prediction models of high-risk infertility. **(A)** Nomogram of Model 1; **(B)** Nomogram of Model 2; **(C)** Nomogram of Model 3. Due to instability or non-significance of the confidence intervals for certain variables in the regression models, the following variables were excluded from the plots: “Occupation: Self-employed” (Model 2), “Occupation: Worker,” and “Contraceptive method: Female sterilization” (Model 3). The coefficients for these variables were automatically excluded from the models and are therefore not displayed in the final nomograms.

**Figure 5:**
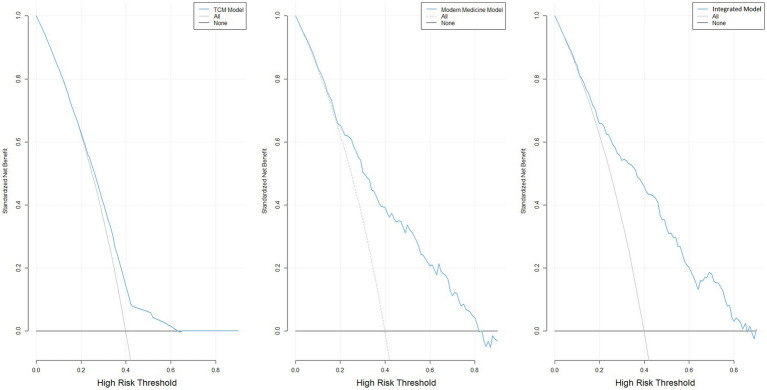
DCA for the prediction models of high-risk infertility. **(A)** Decision curve of Model 1; **(B)** Decision curve of Model 2; **(C)** Decision curve of Model 3.

To facilitate clinical application, nomograms were constructed for Models 1, 2, and 3 (Figure 4) to predict the probability of high-risk infertility for individual patients. The nomograms assign points to each predictive variable and calculate the total score to obtain the corresponding predicted probability. DCA was further employed to assess the clinical net benefit of the three models at different threshold probabilities (Figure 5). The results showed that across a broad range of threshold probabilities, Model 3 achieved an overall net benefit higher than that of Model 1 and comparable to that of Model 2, suggesting that the integration of TCM and modern medical variables did not result in an evident disadvantage in clinical decision-making, although the incremental benefit remained limited. Model 2’s net benefit was between the two, and it outperformed Model 1 in most clinically relevant threshold intervals. Within the low-to-moderate range of threshold probabilities, the net benefit curves of Model 2 and Model 3 were consistently higher than those of the “treat-all” and “treat-none” strategies, indicating that neither model showed a clear disadvantage across the evaluated thresholds. Accordingly, the DCA results should be interpreted primarily as providing exploratory support for risk stratification rather than definitive guidance for clinical decision-making.

In the incremental performance assessment, based on apparent performance metrics, Model 3 showed a slight increase in the AUC compared with Model 2 (*Δ*AUC = 0.02), suggesting a marginal numerical difference in discriminative ability. However, within the threshold probability range of 0.1–0.8, the difference in the area under the net benefit curve (AUNB) derived from decision curve analysis was close to zero (Δ = −0.0022) and inconsistent in direction, indicating limited incremental advantage in terms of clinical net benefit. With respect to overall prediction error, Model 3 yielded a slightly lower Brier score than Model 2 (Δ = −0.0080), although this difference was likewise not consistently reflected in the bias-corrected metrics. Taken together, the inclusion of TCM-related variables did not confer a stable incremental predictive value in the corrected performance evaluation; their contribution appears to lie primarily in exploratory informational augmentation rather than in a robust improvement of overall model performance (Supplementary Table S3).

## 4 Discussion

In this study, we conducted a secondary analysis of 409 women with infertility, aiming to systematically evaluate the role of demographic characteristics, modern medical factors, and TCM syndrome and constitution types in high-risk infertility stratification. The results showed significant differences in several demographic and reproductive-related indicators between the high-risk and non-high-risk groups, suggesting that high-risk infertility status has distinct multidimensional characteristics. In terms of TCM diagnostic features, Qi-deficiency constitution was significantly more prevalent in the high-risk group. In contrast, the proportion of Phlegm-dampness obstruction syndrome was relatively lower, suggesting that different TCM constitutions and syndromes may play distinct roles in risk stratification. Although Kidney-deficiency syndrome was common in both groups, it did not show significant discriminatory ability, suggesting that it is more likely to reflect the underlying constitutional background of infertility.

This study constructed three predictive models based on individual LASSO regression models, with variables selected from different categories. The results showed that the integrative model performed best in terms of discriminative ability, calibration performance, and clinical net benefit. This ensured consistent variable selection and coherent model construction throughout the process. The clinical value of this model does not lie in replicating already observable diagnostic results but in assisting early clinical triage and individualized risk stratification during the dynamic evolution phase of infertility progression and etiology evaluation. This model is particularly designed for situations where a comprehensive diagnostic workup is not yet completed but where there is sufficient clinical information to predict a trajectory toward high-risk infertility. By identifying women whose risk characteristics are similar to those of established high-risk infertility populations, this model can be applied to early infertility patients to guide closer follow-up or timely referral.

### 4.1 Clinical characteristics and TCM pattern recognition of high-risk infertility

As shown in the baseline comparison (Table 1), patients in the high-risk infertility group exhibited distinct clinical characteristics across multiple dimensions. The mean age was 34 years, significantly higher than that of the non-high-risk group (*p* < 0.001), suggesting that age is a critical risk factor, especially after 35, when declines in oocyte quality, oxidative stress, and cell cycle abnormalities accelerate infertility (13, 14). A significant difference in occupational distribution was observed between the high-risk and non–high-risk infertility groups (*p* = 0.00604). Previous studies have suggested that occupational factors are closely associated with infertility risk, particularly through mechanisms related to work intensity, physical workload, shift work, and environmental or occupational exposures (15). Therefore, occupation may not function as a single causal factor but rather as a composite sociodemographic indicator reflecting work-related stress, lifestyle patterns, and potential health risks relevant to reproductive outcomes.

Additionally, the proportion of ‘low sexual activity frequency’ was higher in the high-risk infertility group (*p* = 0.046), which aligns with reproductive medicine common knowledge, as reduced sexual activity frequency may decrease the chances of conception and prolong the duration of infertility. In addition, among reproductive history–related indicators, a statistically significant difference in gravidity distribution was identified between the high-risk and non–high-risk groups (*p* = 0.0482), suggesting that prior pregnancy history may carry discriminative value in infertility risk stratification. Previous evidence indicates that women with no prior pregnancies or lower gravidity exhibit lower natural conception probabilities and a higher risk of infertility across different age groups, highlighting gravidity as not only a descriptive marker of past reproductive outcomes but also a proxy indicator of underlying fecundity (16). Furthermore, the distribution of previous miscarriage numbers also differed significantly between the two groups (*p* = 0.0218). On one hand, an increasing number of prior miscarriages is negatively associated with fecundity, with women experiencing one or more miscarriages demonstrating progressively reduced reproductive potential.

On the other hand, recurrent pregnancy loss may contribute to pelvic inflammation, intrauterine adhesions, or other reproductive tract damage, thereby further impairing the likelihood of successful conception and increasing infertility risk (17, 18). Additionally, recurrent miscarriages may reflect underlying uterine disorders, endocrine dysfunction, or immune abnormalities, further contributing to infertility (19). A significantly higher proportion of women in the high-risk group had undergone prior gynecological surgeries compared to the non-high-risk group (39.3% vs. 24.8%, *p* = 0.0027), which is associated with increased rates of tubal damage and pelvic adhesions (20, 21). Regarding contraceptive methods, the proportion of women who had never used contraception was significantly higher in the high-risk group than in the non–high-risk group (50.3% vs. 31.7%, *p* = 0.001), suggesting that some women experienced prolonged unprotected intercourse without achieving pregnancy and thus inherently met the definition of infertility. In addition, although some individuals had reproductive intention and deliberately avoided contraception, underlying reproductive abnormalities may have prevented successful conception, ultimately leading to classification as high-risk infertility.

### 4.2 Distribution characteristics of TCM syndromes and constitutions in the high-risk infertility population

From the perspective of TCM, the present study revealed that the proportions of Qi-deficiency constitution were significantly higher in the high-risk infertility group. In contrast, the prevalence of Phlegm-dampness obstruction syndrome was notably lower (Tables 2, 3). Phlegm-dampness obstruction syndrome accounted for only 2.5% in the high-risk group, which was significantly lower than 9% in the non–high-risk group (*p* = 0.0148), making it the only TCM syndrome in the current analysis that showed a statistically significant difference between the two groups. This finding indicates that Phlegm-dampness obstruction syndrome is relatively uncommon in the high-risk population, possibly due to its distinct etiological characteristics. Clinically, this syndrome is frequently observed in patients with metabolic or functional infertility, such as those with PCOS, obesity, or insulin resistance (22, 23). These patients typically present with prominent symptoms and well-defined etiologies, often showing excess-type pathogenesis. They tend to respond well to interventions such as dietary adjustment, weight reduction, and pharmacologic treatment. They thus are more likely to have shorter disease durations and simpler etiologies, making them less likely to meet the criteria for high-risk infertility as defined in this study. Notably, previous studies have highlighted the critical role of Kidney-deficiency syndrome in infertility and have gradually elucidated its underlying biological mechanisms, thereby providing molecular-level support for its theoretical foundation (24, 25). In this study, the prevalence of Kidney-deficiency syndrome in the high-risk group was 65.0%, higher than 55.1% in the non-high-risk group, with a marginally significant difference (*p* = 0.0581). This suggests that Kidney-deficiency may be more common among women at high risk of infertility and could serve as a potential warning indicator. However, further validation in larger samples is warranted.

Regarding the TCM constitution, the prevalence of Qi-deficiency constitution in the high-risk group was 12.9%, which was significantly higher than the 5.3% in the non-high-risk group (*p* = 0.0114). This suggests a potential association between Qi-deficiency constitution and the severity of infertility risk. In TCM theory, qi promotes blood generation, and qi deficiency leads to blood weakness, resulting in insufficiency of both qi and blood. This may impair the production of Tian Gui, deplete the Chong and Ren meridians, and ultimately hinder conception (26).

In this study, based on the variables selected by LASSO regression, we further analyzed TCM syndromes and constitution types in Models 1 and 3. Specifically, the Qi-stagnation constitution, the Balanced constitution, and the Stasis obstruction in the uterus syndrome were selected. Among them, the standardized regression coefficient for Stasis obstruction in the uterus syndrome was 0.06 (Table 6). Although its predictive power was relatively weak, it still showed a positive predictive effect when combined with other variables. Blood stasis obstructing the uterine vessels and disharmony of the Chong and Ren meridians prevent the uterus from receiving the essence, ultimately leading to infertility. A systematic review and meta-analysis by Bai et al. (27) shows that TCM treatment based on the theory of “Kidney-deficiency and Blood-stasis,” with tonifying the kidney and activating blood circulation, can significantly improve pregnancy rates in patients with immune infertility. The core mechanism involves improving the blood stasis state in the uterus and regulating reproductive immune function, providing high-level evidence for TCM treatment of Stasis Obstruction in the Uterus Syndrome.

In contrast, the standardized regression coefficients for Qi-stagnation constitution and balanced constitution were −0.032 and −0.055, showing an adverse predictive effect. This suggests that, although the proportion of these two constitution types in the high-risk infertility group is relatively low, their role in the overall model should not be overlooked. The liver is crucial to female reproduction; it stores blood and plays a key role in regulating menstruation and pregnancy. However, infertile patients, due to long treatment cycles, high costs, and the dual pressure of family and society, are prone to negative emotions such as stress and anxiety, leading to Liver Qi Stagnation, which forms a Qi-stagnation constitution. A Qi-stagnation constitution can cause the liver to lose its regulatory function, affecting the Chong and Ren meridians, which, in turn, can impact fertility. Modern medical research also shows that negative emotional states exacerbate infertility, creating a vicious cycle (28). While the Balanced constitution is considered a healthier constitution type, it did not show strong predictive power. However, its inclusion in Model 3 provides comparative information for predicting high-risk infertility, thereby enhancing the model’s accuracy and stability.

### 4.3 Construction and performance evaluation of the risk prediction model

Although this study compared the distribution of TCM syndromes and constitutions between high-risk and non-high-risk infertility groups in Tables 2, 3, only a few TCM diagnostic variables showed statistically significant differences between the groups, indicating that individual TCM syndromes or constitutional features have limited ability to distinguish high-risk infertility when used in isolation. This result reflects the multifactorial nature of infertility risk. It supports placing TCM diagnostic information within a multivariable framework for comprehensive evaluation, rather than using it as an independent judgment criterion. Based on this understanding, this study constructed three models during the modeling phase: a TCM variable model, a modern medical variable model, and an integrated model to explore the relative contributions of different information dimensions in risk stratification. The integrated model included both TCM diagnostic features and clinically available variables, emphasizing the complex background of high-risk infertility from demographic characteristics, reproductive history, menstrual features, and TCM diagnostic perspectives. Overall, the model comparison results suggest that modern clinical and reproductive factors play a dominant role in risk prediction. At the same time, TCM diagnostic information serves primarily as a supplementary feature, providing only a limited improvement in model performance. Notably, the incremental performance analysis in this study shows that including TCM variables contributes very little to improving model performance. Specifically, although Model 3 exhibited a slight improvement over Model 2 in terms of apparent AUC (ΔAUC = 0.02), the differences between the two models in the AUNB derived from decision curve analysis and in overall prediction error (Brier score) were close to zero and inconsistent in direction. This indicates that the inclusion of TCM-related variables did not yield a robust gain in discriminative performance in the bias-corrected evaluation. This suggests that including TCM variables does not significantly improve the model’s discriminative ability. It is important to note that this study does not interpret TCM syndromes or constitutions as direct causes or protective factors for high-risk infertility, but instead considers them as risk representations that may reflect overall physiological status and regulatory capacity. The TCM model’s predictive ability is relatively limited and is better suited to evaluating the potential information in TCM diagnostic data for risk stratification, rather than serving as an independent clinical decision-making tool. By contrast, the integrative model did not demonstrate a clear and stable advantage over the modern medicine model in terms of corrected discrimination performance or net benefit metrics. Its potential clinical utility therefore requires further evaluation through external validation, and any incremental improvement should be interpreted with caution. Additionally, the comparison of model performance differences in this study is mainly based on descriptive metrics (such as AUC, Brier score, and decision curve shape), without formal hypothesis testing or uncertainty interval estimation for ΔAUC or net benefit differences. Therefore, all statements regarding incremental improvements in the models should avoid implying statistical significance and should be clearly stated as exploratory analysis results. It is important to emphasize that the predictive models developed in this study are not intended to replace existing infertility diagnostic processes, nor are they meant to re-evaluate outcomes that have already been clearly established. It should be explicitly stated that, although the definition of high-risk infertility in this study was based on confirmed etiological factors and disease duration, the constructed models were intended to perform risk stratification by estimating the probability that a patient would meet the high-risk classification as clinical information is progressively obtained during the assessment process, rather than to classify patients using etiological information that is already known at the time of decision-making. In this context, the models can assist clinicians in identifying potential high-risk individuals earlier, allowing for more careful decision-making regarding follow-up arrangements, evaluation intensity, and the timing of interventions.

## 5 Strengths and limitations

This study offers methodological and practice-oriented value. First, we proposed an operational definition of high-risk infertility for predictive modeling that integrates disease duration with etiological burden, aiming to capture heterogeneity among infertile women in terms of clinical complexity and cumulative etiological factors, thereby providing a reference framework for risk stratification and follow-up management during clinical assessment. In addition, information on TCM constitution and syndrome differentiation was systematically incorporated into the modeling workflow, enabling the transformation of TCM diagnostic information into structured and quantifiable variables and providing a reproducible technical pathway for integrating TCM informatics with modern risk prediction methods. With respect to modeling strategy, given the relatively large number of candidate predictors and the limited number of outcome events, LASSO penalized regression was adopted for variable selection and model construction to reduce the effective degrees of freedom through coefficient shrinkage and to mitigate the risk of overfitting, with the penalty parameter determined via ten-fold cross-validation. On this basis, internal validation and optimism correction were further performed using bootstrap resampling, combined with out-of-bag evaluation, to quantify predictive bias and uncertainty in discrimination, calibration, and overall prediction error under a fixed model structure. Notably, after resampling-based correction, no extreme imbalance was observed in discrimination performance, calibration slopes, or Brier scores across the models, suggesting that, under the current data conditions and modeling framework, the overall model performance retains a degree of structural interpretability. These findings support the use of the proposed models as exploratory and comparative tools for risk stratification, aimed at characterizing the relative contributions of different variable domains to the identification of high-risk infertility. At the same time, they indicate that, given the present sample size and variable complexity, more complex integrative models may be subject to greater overfitting penalties and therefore should not be interpreted as definitive clinical decision-making tools.

Nevertheless, this study has several limitations. First, it is based on single-center, cross-sectional data and has not yet undergone external validation in multicenter settings or across diverse populations; thus, the generalizability of the models remains to be further evaluated. Although internal validation using bootstrap resampling with optimism correction was performed, the corrected results indicated that the integrative model did not consistently outperform the modern medicine model in terms of discrimination or calibration. This suggests that the observed performance gains may be sensitive to sample characteristics and modeling conditions, and that, in the absence of independent external validation cohorts, the generalizability of the models requires further confirmation in other populations. Second, some key variables—such as TCM constitution, TCM syndrome patterns, and sexual activity frequency—were obtained through self-report, and are therefore inevitably subject to recall bias and inter-individual differences in subjective judgment. In addition, although all participants underwent routine infertility evaluation, the specific scope and depth of diagnostic investigations may have varied according to prior healthcare-seeking history and clinical decision-making, potentially introducing diagnostic confirmation bias into the outcome component of “multiple etiologies of infertility.” Third, the predictive models did not incorporate specific modern reproductive indicators, such as detailed hormonal profiles, follicular monitoring data, or advanced imaging parameters, which limits the depth of biological interpretation. Future studies should adopt prospective designs with independent multicenter cohorts and further integrate TCM diagnostic information, modern reproductive indicators, and psychological factors to develop more robust, clinically applicable multidimensional risk assessment models.

## 6 Conclusion

In summary, this study developed and compared different types of predictive models for high-risk infertility based on multidimensional information from both TCM and modern medicine. After internal validation and optimism correction, the modern medicine model demonstrated more robust predictive performance in terms of discrimination, calibration, and overall prediction error. Although the integrative model incorporating TCM differentiation information showed a numerical improvement in apparent discriminative ability, this advantage was not consistently retained after correction, suggesting that under the current sample size and model complexity, the inclusion of additional variables may be subject to overfitting penalties. Importantly, the predictive models developed in this study are not intended to replace existing diagnostic criteria for high-risk infertility, but rather to serve as supportive tools for risk stratification during the infertility care pathway, particularly for women in whom etiological evaluation is still ongoing but who already exhibit multidimensional high-risk features. In this context, the value of TCM differentiation variables lies primarily in providing complementary, structured information for risk stratification, rather than acting as independent or dominant predictors. This study offers a methodological exploration of the structured application of TCM differentiation information in infertility risk assessment and provides practical evidence for optimizing risk stratification and clinical decision support within an integrative Chinese–Western medicine framework. The clinical applicability and incremental value of the proposed models still require further evaluation in larger samples and through external validation studies.

## Data Availability

The original contributions presented in the study are included in the article/Supplementary material, further inquiries can be directed to the corresponding author/s.

## References

[1] Carson SA Kallen AN. Diagnosis and management of infertility: a review. JAMA. (2021) 326:65–76. doi: 10.1001/jama.2021.4788, 34228062 PMC9302705

[2] Vander Borght M Wyns C. Fertility and infertility: definition and epidemiology. Clin Biochem. (2018) 62:2–10. doi: 10.1016/j.clinbiochem.2018.03.012, 29555319

[3] Oluwaseun OM Kunle AM John OO. ICT adoption for Marketing in the Nigerian Paints Industry. The Ninth International Conference on Applications of Information Communication Technologies to Teaching, Research and Administration; 2014. Ilorin, Nigeria: AICTTRA Organizers (Obafemi Awolowo University in collaboration with Kwara State University).

[4] Qi FY. Association between Chinese medicine body constitution and variants in metabolic gene (Rs1501299 & Rs1801282) Universiti Tunku Abdul Rahman (2022).

[5] Yu R Zhao X Li L Ni C Yang Y Han Y . Consistency between traditional Chinese medicine constitution-based classification and genetic classification. J Tradit Chin Med Sci. (2015) 2:248–57. doi: 10.1038/s41598-025-26439-6

[6] Cheng F Wang X Song W Lu Y Li X Zhang H . Biologic basis of Tcm syndromes and the standardization of syndrome classification. J Tradit Chin Med Sci. (2014) 1:92–7. doi: 10.1016/j.jtcms.2014.09.005

[7] Zhuwei G Xiaoling M Yixiao H Jiannan Y Jiaxing F Yang L . Clinical study on the correlation between syndrome types and traditional Chinese medicine constitution in 409 cases of female infertility. Adv Integr Med. (2025) 12:100579. doi: 10.1016/j.aimed.2025.100579

[8] Kong BH Ma D Duan T Di W Zhu L Qi HB Obstetrics and Gynecology. 10th Edn. Beijing: People's Medical Publishing House (2024) pp. 396–398.

[9] Zhang YZ. Traditional Chinese Medicine Gynecology (2nd Edn., National Planning Textbook for the twelfth five-year plan of higher education) Beijing: China Press of Traditional Chinese Medicine (2014).

[10] Yang YH Huang GN Sun HX Fan LQ Feng Y Shen H . Chinese expert consensus on the diagnosis and treatment of unexplained infertility. J Reprod Med. (2019) 28:984–92. doi: 10.3969/j.issn.1004-3845.2019.09.002

[11] Infertility Guideline Group of the European Society of Human Reproduction and Embryology (ESHRE), Romualdi D Ata B Bhattacharya S Bosch E Costello M Evidence-Based Guideline: Unexplained Infertility. Human Reproduction (2023) 38:1881–1890. doi: 10.1093/humrep 37599566 PMC10546081

[12] National Collaborating Centre for Women’s and Children’s Health (UK). Fertility: Assessment and treatment for people with fertility problems. London: Royal College of Obstetricians & Gynaecologists, 2013. Available online at: https://pubmed.ncbi.nlm.nih.gov/25340218/25340218

[13] Tatone C. Oocyte senescence: a firm link to age-related female subfertility. Gynecol Endocrinol. (2008) 24:59–63. doi: 10.1080/09513590701733504, 18210326

[14] García D Brazal S Rodríguez A Prat A Vassena R. Knowledge of age-related fertility decline in women: a systematic review. Eur J Obstet Gynecol Reprod Biol. (2018) 230:109–18. doi: 10.1016/j.ejogrb.2018.09.030 30248536

[15] Abdoli S Masoumi SZ Kazemi F. Environmental and occupational factors and higher risk of couple infertility: a systematic review study. Middle East Fertil Soc J. (2022) 27:33. doi: 10.1186/s43043-022-00124-4

[16] Steiner AZ Jukic AMZ. Impact of female age and nulligravidity on fecundity in an older reproductive age cohort. Fertil Steril. (2016) 105:1584–8. e1. doi: 10.1016/j.fertnstert.2016.02.028, 26953733 PMC4893975

[17] Arge LA Håberg SE Wilcox AJ Næss Ø Basso O Magnus MC. The association between miscarriage and fecundability: the Norwegian mother, father and child cohort study. Hum Reprod. (2022) 37:322–32. doi: 10.1093/humrep 34792121 PMC8804331

[18] Pike GK. Abortion and infertility. Issues L Med. (2020) 35:173. doi: 10.1186/s43043-022-00124-4 33950600

[19] Wang H-Y Qiao J Sun X-X Wang S-Y Liang X-Y Sun Y . Epidemiological survey and risk factor analysis of recurrent spontaneous miscarriages in infertile women at large infertility centers. Chin Med J. (2016) 130:2056–62. doi: 10.4103/0366-6999.213415 PMC5586173 28836548

[20] Ahmed RN. Radiological evaluation of uterine tubes in infertile women with previous pelvic surgery. Med J Babylon. (2024) 21:369–74. doi: 10.4103/MJBL.MJBL_904_23

[21] Koninckx PR Ussia A Adamyan L Gomel V. Reproductive surgery in the 21st century. Glob Reprod Health. (2018) 3:e12. doi: 10.1097/GRH.0000000000000012

[22] Luo H Li L Li T Liao X Wang Q. Association between metabolic syndrome and body constitution of traditional Chinese medicine: a systematic review and meta-analysis. J Tradit Chin Med Sci. (2020) 7:355–65. doi: 10.1016/j.jtcms.2020.10.004

[23] Abadi F Yee L Month L Ting D Suan S. The analysis of Chinese medicine body constitutions of polycystic ovarian syndrome patients in Malaysia. J CAM Research Progress. (2023) 2:110. doi: 10.33790/jcrp110011

[24] Tang N Liu L Qiu H Shi W Mao D. Analysis of gene expression and functional changes of adrenal gland in a rat model of kidney Yang deficiency syndrome treated with Sini decoction. Exp Ther Med. (2018) 16:3107–15. doi: 10.3892/etm.2018.6521, 30214533 PMC6125868

[25] Liu D-Q Wei C-F Zhang X Xiang S Lian F. Microrna profiling reveals effects of Erzhi Tiangui granules on kidney deficiency diminished ovarian reserve: a randomized trial. Medicine (Baltimore). (2023) 102:e33652. doi: 10.1097/MD.0000000000033652, 37115053 PMC10145740

[26] Mei RB Zhang YC Zheng YW. Constitution distribution characteristics of 922 infertility patients in traditional Chinese medicine. China J Tradit Chin Med Pharm. (2024) 39:3798–801.

[27] Bai Y-l Chen Y-h Jiang C Qian J-h Han L-l Lu H-z . Efficacy and safety of traditional Chinese medicine in the treatment of immune infertility based on the theory of “kidney deficiency and blood stasis”: a systematic review and meta-analysis. Evid Based Complement Alternat Med. (2021) 2021:9947348. doi: 10.1155/2021/9947348 34055028 PMC8149227

[28] Zhang R Chen C Tan J Liu J Fan Q. Influencing factors of infertility with Qi-stagnation constitution in the Ludong area. Guangming J Chin Med. (2025) 40:1253–6. doi: 10.3969/j.issn.1003-8914.2025.07.001

