# Supplementary material for: Development of a predictive model for high-risk infertility based on TCM constitution and syndrome: a secondary analysis
Source: Front Med (Lausanne). 2026 Feb 18;13:1738707. doi: 10.3389/fmed.2026.1738707 (PMC12956622; doi:10.3389/fmed.2026.1738707)
Supplement: Supplementary file 1 [file Data_Sheet_1.docx]

**Supplementary Figure S1. Forest plot of risk factors associated with high-risk infertility**


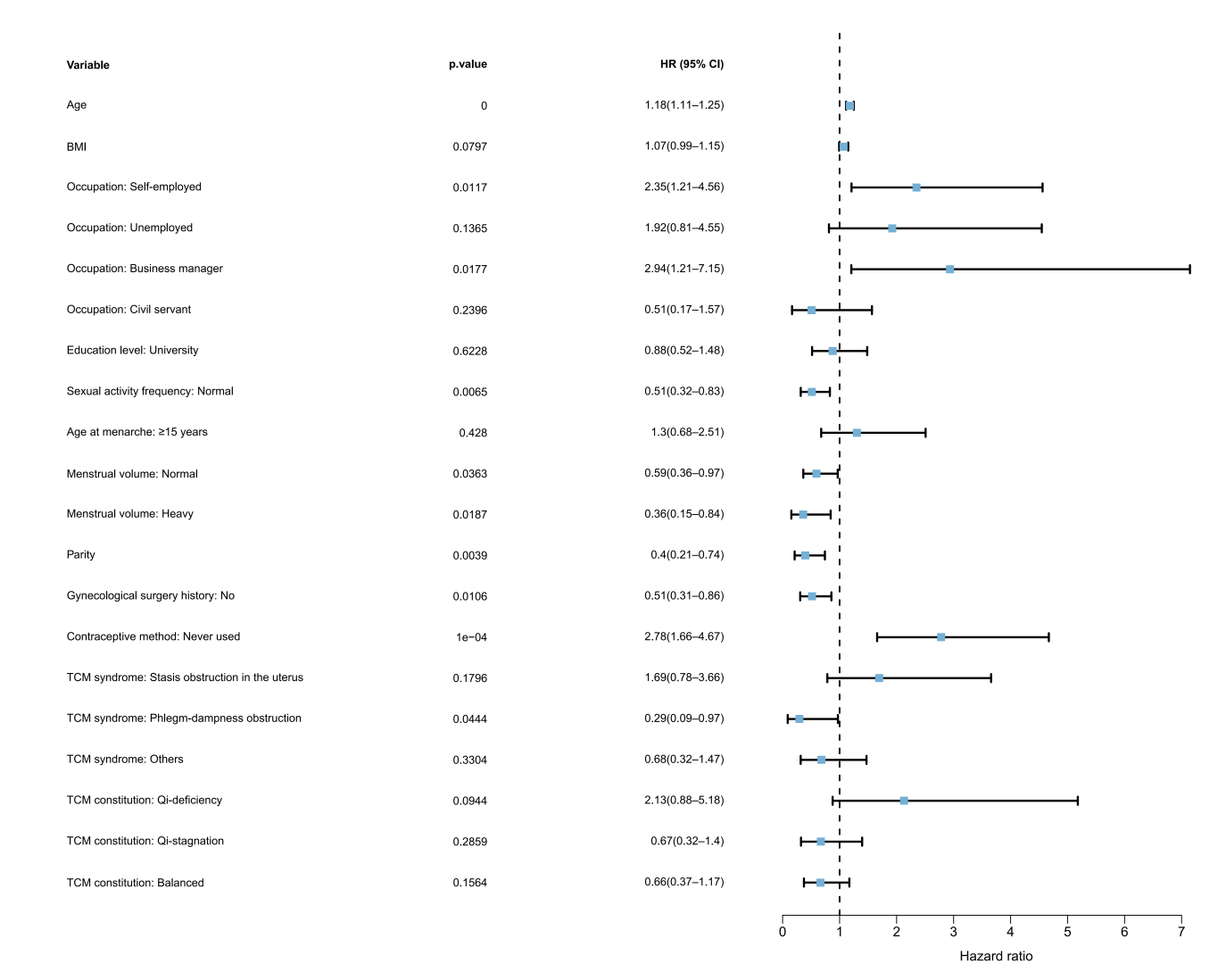


**Supplementary Table S1. Missing Data Summary of Study Variables (N = 409)**

| Variable | Valid (N) | Missing (N) | Missing (%) |
| --- | --- | --- | --- |
| Age | 406 | 3 | 0.7 |
| Ethnicity | 409 | 0 | 0 |
| BMI | 407 | 2 | 0.5 |
| Occupation | 398 | 11 | 2.7 |
| Education level | 409 | 0 | 0 |
| Sexual activity frequency | 409 | 0 | 0 |
| Age at menarche | 374 | 35 | 8.6 |
| Menstrual duration | 404 | 5 | 1.2 |
| Menstrual cycle length | 406 | 3 | 0.7 |
| Menstrual volume | 409 | 0 | 0 |
| Gravidity | 409 | 0 | 0 |
| Parity | 409 | 0 | 0 |
| Number of miscarriages | 407 | 2 | 0.5 |
| Infertility duration (years) | 409 | 0 | 0 |
| History of gynecological surgery | 409 | 0 | 0 |
| Contraceptive method | 409 | 0 | 0 |
| Kidney-deficiency syndrome | 409 | 0 | 0 |
| Liver-depression and Qi-stagnation syndrome | 409 | 0 | 0 |
| Stasis obstruction in the uterus syndrome | 409 | 0 | 0 |
| Phlegm-dampness obstruction syndrome | 409 | 0 | 0 |
| Others | 409 | 0 | 0 |
| Yang-deficiency constitution | 409 | 0 | 0 |
| Yin-deficiency constitution | 409 | 0 | 0 |
| Qi-deficiency constitution | 409 | 0 | 0 |
| Phlegm-dampness constitution | 409 | 0 | 0 |
| Damp-heat constitution | 409 | 0 | 0 |
| Blood-stasis constitution | 409 | 0 | 0 |
| Special constitution | 409 | 0 | 0 |
| Qi-stagnation constitution | 409 | 0 | 0 |
| Balanced constitution | 409 | 0 | 0 |
| Ovarian factor | 409 | 0 | 0 |
| Tubal factor | 409 | 0 | 0 |
| Uterine factor | 409 | 0 | 0 |
| Immune factor | 409 | 0 | 0 |
| Unexplained infertility | 409 | 0 | 0 |

**Supplementary Table S2. Variable Coding Scheme, Reference Categories, and Recommended Reporting Format for Multivariable Logistic Regression Analysis**

| Variable | Levels shown in manuscript/output | Reference level (used for dummy coding) | How to report in tables (recommended) |
| --- | --- | --- | --- |
| Age | Continuous (years) | Not applicable (continuous) | OR per 1-year increase |
| Ethnicity | Han; Minority | Minority | Han vs Minority (ref) |
| BMI | Continuous (kg/m²) | Not applicable (continuous) | OR per 1-unit increase |
| Occupation | General Employee; Other categories | General Employee | Each occupation vs General Employee (ref) |
| Education level | Junior high or below; High school; Junior college; University; Postgraduate | Junior high or below | Other levels vs Junior high or below (ref) |
| Sexual activity frequency | Low; Normal | Low | Normal vs Low (ref) |
| Age at menarche | ≤12 years; 13–14 years; ≥15 years | ≤12 years | 13–14 vs ≤12 (ref); ≥15 vs ≤12 (ref) |
| Menstrual duration | ≤2 days; 3–7 days; ≥8 days | ≤2 days | 3–7 vs ≤2 (ref); ≥8 vs ≤2 (ref) |
| Menstrual cycle length | ≤20 days; 21–35 days; ≥36 days | ≤20 days | 21–35 vs ≤20 (ref); ≥36 vs ≤20 (ref) |
| Menstrual volume | Light; Normal; Heavy | Light | Normal vs Light (ref); Heavy vs Light (ref) |
| Gravidity | Continuous (count) | Not applicable (continuous) | OR per 1-pregnancy increase |
| Parity | Continuous (count) | Not applicable (continuous) | OR per 1-birth increase |
| Number of miscarriages | Continuous (count) | Not applicable (continuous) | OR per 1-miscarriage increase |
| History of gynecological surgery | Yes; No | Yes | No vs Yes (ref) |
| Contraceptive method | IUD; Emergency contraception; Female sterilization; Hormonal contraception; Condom; Never used | IUD | Each method vs IUD (ref) |
| Kidney-deficiency syndrome | Yes; No | No | Yes vs No (ref) |
| Liver-depression and Qi-stagnation syndrome | Yes; No | No | Yes vs No (ref) |
| Stasis obstruction in the uterus syndrome | Yes; No | No | Yes vs No (ref) |
| Phlegm-dampness obstruction syndrome | Yes; No | No | Yes vs No (ref) |
| Others (TCM syndrome) | Yes; No | No | Yes vs No (ref) |
| Yang-deficiency constitution | Yes; No | No | Yes vs No (ref) |
| Yin-deficiency constitution | Yes; No | No | Yes vs No (ref) |
| Qi-deficiency constitution | Yes; No | No | Yes vs No (ref) |
| Phlegm-dampness constitution | Yes; No | No | Yes vs No (ref) |
| Damp-heat constitution | Yes; No | No | Yes vs No (ref) |
| Blood-stasis constitution | Yes; No | No | Yes vs No (ref) |
| Special constitution | Yes; No | No | Yes vs No (ref) |
| Qi-stagnation constitution | Yes; No | No | Yes vs No (ref) |
| Balanced constitution | Yes; No | No | Yes vs No (ref) |

**Supplementary Table S3. Apparent incremental performance metrics comparing Model 3 and Model 2**

| Evaluation Indicator | Model 2 | Model 3 | Δ (Model 3 − Model 2) |
| --- | --- | --- | --- |
| AUC | 0.771 | 0.791 | +0.02 |
| AUNB | 1.7445 | 1.7423 | −0.0022 |
| Brier Score | 0.1873 | 0.1793 | −0.0080 |

Note: The AUNB was calculated using the trapezoidal rule across threshold probabilities from 0.1 to 0.8, based on standardized net benefit. All values are based on apparent (uncorrected) performance estimates. Incremental differences were not preserved after optimism correction.
